# Macro-Invertebrate Decline in Surface Water Polluted with Imidacloprid: A Rebuttal and Some New Analyses

**DOI:** 10.1371/journal.pone.0089837

**Published:** 2014-02-28

**Authors:** Martina G. Vijver, Paul J. van den Brink

**Affiliations:** 1 Institute of Environmental Sciences (CML), Leiden University, Leiden, The Netherlands; 2 Alterra, Wageningen University and Research centre, Wageningen, The Netherlands; 3 Wageningen University, Wageningen University and Research centre, Wageningen, The Netherlands; Texas Tech University, United States of America

## Abstract

Imidacloprid, the largest selling insecticide in the world, has received particular attention from scientists, policymakers and industries due to its potential toxicity to bees and aquatic organisms. The decline of aquatic macro-invertebrates due to imidacloprid concentrations in the Dutch surface waters was hypothesised in a recent paper by Van Dijk, Van Staalduinen and Van der Sluijs (PLOS ONE, May 2013). Although we do not disagree with imidacloprid's inherent toxicity to aquatic organisms, we have fundamental concerns regarding the way the data were analysed and interpreted. Here, we demonstrate that the underlying toxicity of imidacloprid in the field situation cannot be understood except in the context of other co-occurring pesticides. Although we agree with Van Dijk and co-workers that effects of imidacloprid can emerge between 13 and 67 ng/L we use a different line of evidence. We present an alternative approach to link imidacloprid concentrations and biological data. We analysed the national set of chemical monitoring data of the year 2009 to estimate the relative contribution of imidacloprid compared to other pesticides in relation to environmental quality target and chronic ecotoxicity threshold exceedances. Moreover, we assessed the relative impact of imidacloprid on the pesticide-induced potential affected fractions of the aquatic communities. We conclude that by choosing to test a starting hypothesis using insufficient data on chemistry and biology that are difficult to link, and by ignoring potential collinear effects of other pesticides present in Dutch surface waters Van Dijk and co-workers do not provide direct evidence that reduced taxon richness and abundance of macroinvertebrates can be attributed to the presence of imidacloprid only. Using a different line of evidence we expect ecological effects of imidacloprid at some of the exposure profiles measured in 2009 in the surface waters of the Netherlands.

## Introduction

The Netherlands is one of the world’s foremost agricultural producers, with 2/3 of the total land mass devoted to agriculture or horticulture. Land use is highly intensive in terms of output per hectare or head of livestock [2]. To achieve such high outputs a vast range of agricultural chemicals are used, including fertilizers, veterinary drugs, pesticides and biocides. Different pesticides are used depending on the crop that is grown on the land. There are several routes that pesticides may enter surface waters. Pesticides may be washed into ditches and rivers by rainfall; surface waters can be contaminated by direct overspray or via runoff and leaching from agricultural fields [3]. Emission to surface waters (and thus pesticide residue concentrations) is dictated by many factors such as distance of the crop from the ditch and the mode of application, weather conditions and so on.

Neonicotinoids are the first new class of insecticides to be introduced in the last 50 years. The neonicotinoid imidacloprid is currently one of the most widely used insecticides in the world [4]. Recently, imidacloprid has received much negative attention: The use of certain neonicotoids has been restricted in some countries due to evidence of an unacceptably high risk of toxicity to bees, but this restriction was not in effect in the Netherlands at the time of writing this paper. On April 29, 2013, the European Union passed a two-year ban on the use of three neonicotinoids: European law restricts the use of imidacloprid, clothianidin, and thiamethoxam on flowering plants for two years unless compelling evidence comes out that proves that the use of the chemicals is environmentally safe [5]. This ban is partially, restricted to some applications in specific crops and likely covers 15% of the total use of the three neonicotinoids in the Netherlands [6]. Temporary suspensions had previously been enacted in countries such as France, Germany, Switzerland and Italy. In March 2013, a review of 200 studies on neonicotinoids was published by Mineau and Palmer [7], calling for a ban on neonicotinoid use as seed treatments because of their toxicity to birds, aquatic invertebrates, and other wildlife. The EPA – USA is now re-evaluating the safety of neonicotinoids.

Van Dijk and co-workers [1] aimed to assess the specific relationship between imidacloprid residues in Dutch surface waters, and the abundance of non-target macro-invertebrate taxa. As also stated by the authors, finding a statistical relationship between those two datasets does not necessarily reflect causality, because there could be other factors (e.g. other pesticide residues, other local habitat factors) which drive observed patterns of abundance. We have some fundamental criticisms on the way the data were analysed and the results were interpreted, and we feel that this can be challenged by existing data. Therefore as a response to the paper of Van Dijk et al [1], and by using additional data, we explore their two key assumptions: 1) residues of pesticides other than imidacloprid, that are collinear with imidacloprid exposure either do not exist or have negligible effects on macroinvertebrate abundance and 2) that imidacloprid concentrations can be extrapolated successfully over 160 days and at a 1 km^2^ spatial scale.

## Materials and methods

### Data collection and treatment

Data on pesticides concentrations in surface water in the Netherlands were obtained from the Dutch Pesticides Atlas. [8]. This is an online tool from which Dutch monitoring data can be collected and processed into a graphic format. Here, data of all pesticide active ingredients and metabolites (n  =  634) collected in 2009 were used, since this data set is contiguous with the data used by Van Dijk et al. [1]. Only one year was selected since it can be expected that the correlations between pesticide occurrences will be year-specific, so this correlation should also be assessed for each year specifically. The 2009 dataset covered 302111 individual measurement records of which 19693 measurements exceeded the reporting limit (LOR). The measurements were performed on 4816 samples obtained from 723 different locations. The sample by pesticide dataset is characterised by missing values (90% of entries) and below LOR values (9% of all entries). This is a result of the fact that every water manager has his own suite of pesticides that is sampled, measured and evaluated. The selection of this suite of pesticides is based on the crops and land-use in their region. This selection of pesticides to be monitored improves the efficiency of the monitoring efforts of the individual water managers but yields a data set that has missing values and with many < LOR values when the data of multiple water managers are combined into one. To obtain frequency distributions of the imidacloprid concentrations, data from 2010 and 2011 have also been used.

Environmental quality standards (EQS) of all pesticides were as follows: for imidacloprid the annual average-EQS value (AA-EQS) is 0.067 µg/L (database value set 2-6-2010), and the maximum allowable concentration (MAC-EQS) is 0.2 µg/L (database value set 2-6-2010) as specified by the European Water Framework Directive. In addition, in the Netherlands, the maximum permissible concentration (MPC) of 0.013 µg/L is an important additional criterion (database value set 8-10-2008).

For all samples in which a pesticide could not be detected or quantified, the database substitutes a value of lower than the LOR. The values of reporting limits vary across samples (unique location x time). In our calculations these measurements below LOR are set as zero. We chose to do so, as choosing any other value below LOR would be arbitrary. Moreover, if not taking zero as a value, any other chosen value will result in relatively high toxicity at intensively measured surface waters even if the pesticides are not applied in that area since all measurements results in a lowest value possible of being below the LOR. These types of assumptions are inherent when working with data sets based on monitoring efforts.

### Collinearity of imidacloprid concentrations with concentrations of other pesticides

Collinearity refers to a linear relationship between two explanatory variables, meaning that one can be linearly predicted from the others with a non-trivial degree of accuracy. Collinearity was determined on the data set of 2009 measurements restricted to all samples with at least one measurement above the LOR. The reduced data contained measured values for 18% of the samples, of which 8% of the total were measurements above the LOR. In order to assess the correlation between the concentrations of different pesticides we needed a sample by pesticide matrix with as little missing values as possible. From this gappy database, the largest closed data sets were extracted using Principal Component Analysis [9]. For this, measured values in the database were coded as one and missing data by zero. After running the PCA, the species-by-substance matrix was sorted, based on the scores of the substances and samples on the first principal component. Using this approach, it was possible to extract closed data sets by extracting groups of samples with the same score on the first principal component. Four data sets could be extracted that contained more than 100 samples in which the same pesticides were measured. One data set did not include imidacloprid and was not taken into account. The remaining three matrices contained 114, 108 and 191 samples, 27, 51 and 54 pesticides, with 11, 11 and 13% of the measurements above the LOR for data set 1, 2 and 3, respectively. All sampling points of data set 1 were within the provinces of Utrecht and Gelderland while all sampling points of data set 2 and 3 were located in the province of South Holland.

The log((1000 * conc) +1) transformed pesticide concentration values were analysed with Principal Component Analysis (PCA) using the Canoco5 computer programme [10], (see Zafar et al. [11] for the rationale of the transformation]. The pesticide data were centred and standardised for each pesticide. The graphical pictures based on orthogonal coordinate systems describe optimal variance in a dataset. Points that are clustered near each other have a strong correlation. PCA [9] transforms data to a new coordinate system such that the greatest variance by any projection of the data comes to lie on the first coordinate (called the first principal component), the second greatest variance on the second coordinate [12].

### Calculating multi substance PAF

**Figure 1:**
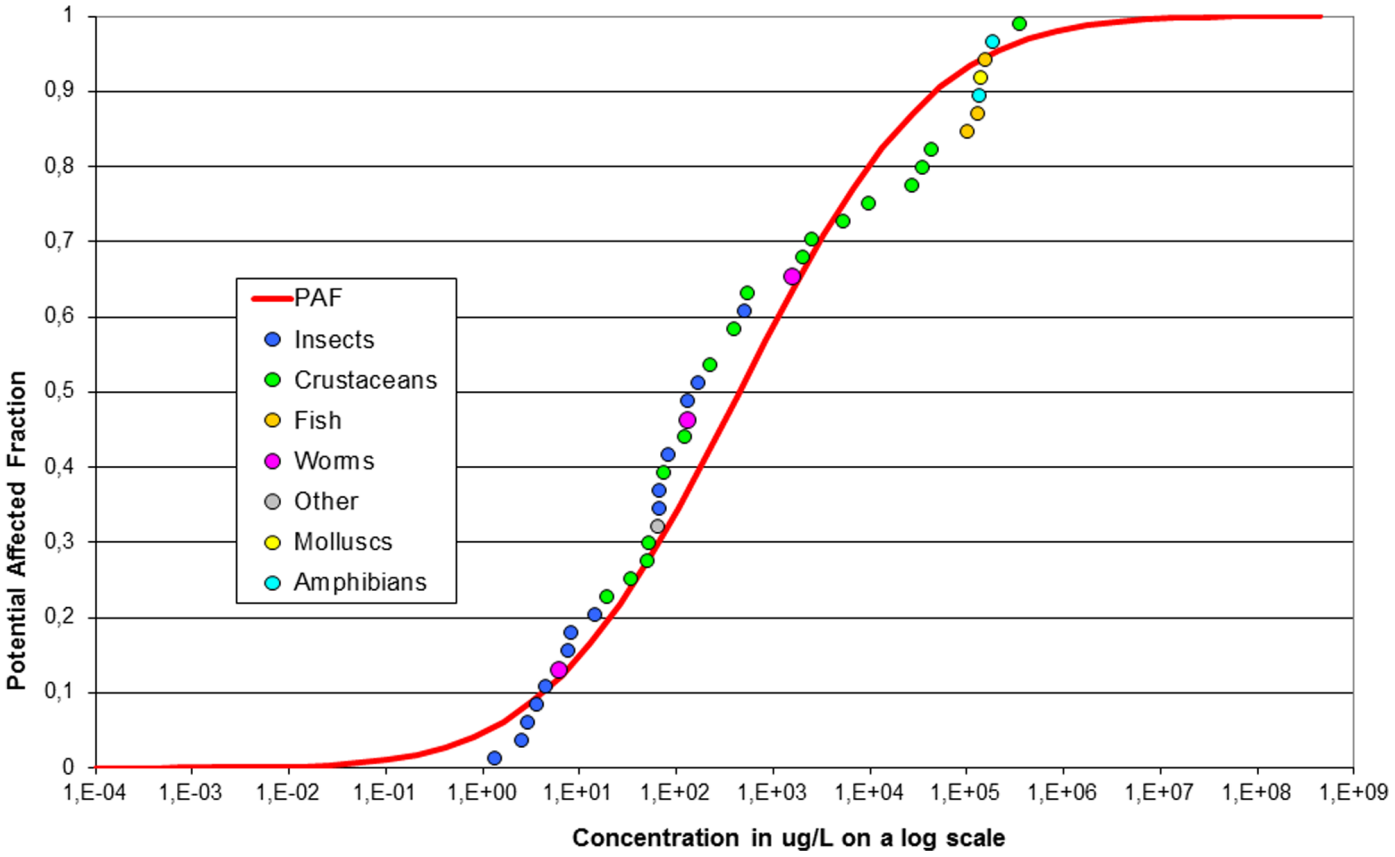
The Species Sensitivity Distribution of imidacloprid based on acute toxicity data. The data consist of 7 different taxomonic groups and 41 species. EPA database downloaded at Oct 23th 2013.

The potential affected fraction (PAF) is a common way to express ecotoxicological risks [13]. Following this approach, measured pesticides concentrations were translated into PAF using the species sensitivity distribution (SSD) approach. Toxicity data for each pesticide was obtained from De Zwart [14], and based on acute median effect concentrations (EC50) as derived in the laboratory (database eTox, RIVM as described in [14]). The eTox database consists mainly of data entries from the ECOTOX EPA database. The SSD for imidacloprid is given in Figure 1, and includes 41 different species from 7 different taxonomic groups. Underlying data including references are given in Table S1 of the Supplementary Information. The full database used for the multi substance PAF (msPAF) calculations contained data of 496 different pesticides with 75 different modes of action. To quantify the ecological impacts due to imidacloprid concentrations amongst all other pesticide concentrations as measured in the surface waters, the msPAF was calculated. Firstly, all concentrations of individual pesticides measured over one month per location were aggregated using the maximum measured value. Secondly individual pesticide concentrations were compared to the toxicity data resulting in the PAF. Thirdly, pesticides were grouped based on their mode of action. The PAF’s of the pesticides with a similar mode-of-action were added using a concentration addition equation. In this equation, each substance concentration is divided by its effect concentration, ECxa, i.e., the concentration of a that represents a standard effect expressed as EC50 for endpoint x. This gives: Emix (Cmix)  =  (Ca/ECxa) + (Cb/ECxb) + …... In which Emix(Cmix) is the summed ratio of the mixture components at the exposure concentration of each chemical (Cx). Fourthly, the different pesticides groups with dissimilar mode-of-action were added using a response addition equation. In response addition, the toxicity of the substances in the mixture can be predicted from the product of the fractional effects of the mixture components. This gives Emix (Cmix)  =  1 – ((1 – E(Ca)) * (1 – E(Cb)) * …... In which Emix(Cmix) is the calculated effect of the mixture, Ca the exposure concentration of substance a, and E(ca) the effect of substance a at concentration Ca.

Both models for mixture toxicity are described in Hewlett and Plackett [15]. Chemicals with an unknown mode-of-action were treated according to a unique mode-of-action. As a result an msPAF value per month per monitoring location was derived. In this study we reported the maximum msPAF of the year 2009. The quantification of the relative contribution of imidacloprid on the total chemical pressure as expressed by msPAF was based on acute toxicity data as insufficient chronic toxicity data were available in the literature.

### Pairwise combinations of samples taken within 1 km and 160 days

Datasets on imidacloprid concentrations and abundances of macroinvertebrates were linked to each other by Van Dijk and co-workers [1] by using the criteria ≤1 km distance and ≤ 160 days of time difference. We performed pairwise comparisons of imidacloprid measurements to determine whether imidacloprid concentrations at sites that meet these criteria, matched successfully. Therefore, all imidacloprid measurements were extracted from the 2009 data set. All sampling sites were first ranked on their x coordinate and the difference in distance with the next sample was assessed (using Pythagoras theorem). All site combinations which yielded a difference less than 1 km were extracted. The same procedure was performed using a ranking based on the y-coordinate. The site combinations from both queries were combined. This procedure is not exhaustive since two sites that are not ranked next to each other can also be closer to 1 km from each other, but is likely to find most combinations. The imidacloprid concentrations of all samples taken at the paired sites were compared to each other when the samples were taken within 160 days. The result of the comparison were categorised into: 1) two measurements below the LOR, 2) one measurement below and one above the LOR (0% matching), 3) two measurements above the LOR, of which the number of sample pairs that matched 100% (based on one decimal) was also noted. The analysis resulted in 37 pairs of sites containing a total of 260 observations and 584 concentration measurement pairs being evaluated.

### Time series of imidacloprid exposure

For each sampling site it was determined how often imidacloprid samples were analysed. For 34 sampling sites 10 or more samples were analysed, of which imidacloprid was not detected in any of the samples at 14 sites (41%), and in less than half of the samples at 28 sites (82%). The concentration dynamics of the remaining 18% of the sites were plotted to evaluate whether chronic concentrations of imidacloprid may be expected.

### Cumulative frequency of maximum imidacloprid concentrations

The measured maximum concentration of each site was compared with threshold concentrations based on the findings of Roessink et al. [16], i.e. the chronic EC10 of the mayfly species *Caenis horaria* and *Cloeon dipterum* (≈0.03 µg/L) and the different environmental quality standards. In order to remove the within-site sample dependency, for each sampling site the maximum imidacloprid concentration was extracted. The analysis resulted in 225 negative measurements (below the LOR) and 226 positive measurements (above the LOR).

### MPC exceedances of imidacloprid compared to other pesticides

Since only for a restricted number of pesticide AA-EQS and MAC-EQS values have been set in the WFD, we used the (Dutch) MPC standard to compare exceedance frequencies between pesticides. For this comparison, both the magnitude of exceedance as well as the frequency of exceedance was incorporated. Firstly, the exceedance of the MPC of an individual pesticide concentration was derived per measuring location. Secondly, the degree of standard exceedance was weighted according to the following classes: 0 (≤MPC); 1 (> MPC and ≤ 2 x MPC), 2 (> 2 x MPC and ≤ 5 x MPC) and 5 (> 5 x MPC exceedance). Thirdly, the exceedance classes were summed over all measuring locations per year. Fourthly, pesticides were ranked on the basis of the weighted number of monitoring sites at which the MPC for the compound was exceeded, i.e. corrected for the number of monitoring sites by taking the percentage of sites that show an exceedance of the MPC. Compounds monitored at fewer than ten sites were ignored.

## Results and Discussion

**Figure 2:**
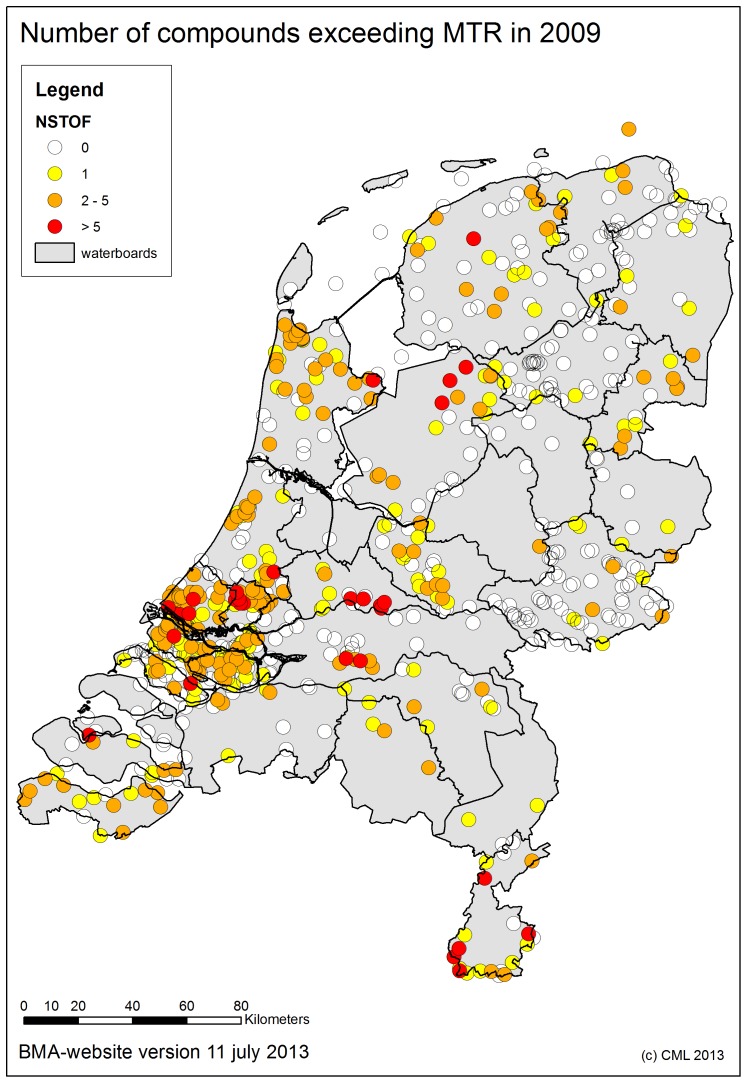
Number of pesticides exceeding the MPC in 2009. All monitoring locations in the Dutch surface waters with one (yellow); two till five pesticides concentrations (orange); and > five different pesticides (red) exceeding their MPC-values are depicted. Locations were measurements were performed but no exceedances were found are depicted in white.

For many locations pesticide concentrations have been found to exceed the MPC in 2009 (see Fig. 2). Figure 2 shows that throughout the entire country more than one pesticide exceeds their respective quality standard, so this exceedance is not a common regionally problem. The maximum amount of pesticides exceeding their MPC in one sample is 35. From this it can be concluded that a single pesticide is not likely to drive solely the macro-invertebrate quality, rather all pesticides exceeding the quality standards should be considered.

### Collinearity of imidacloprid concentrations with other pesticides

**Figure 3:**
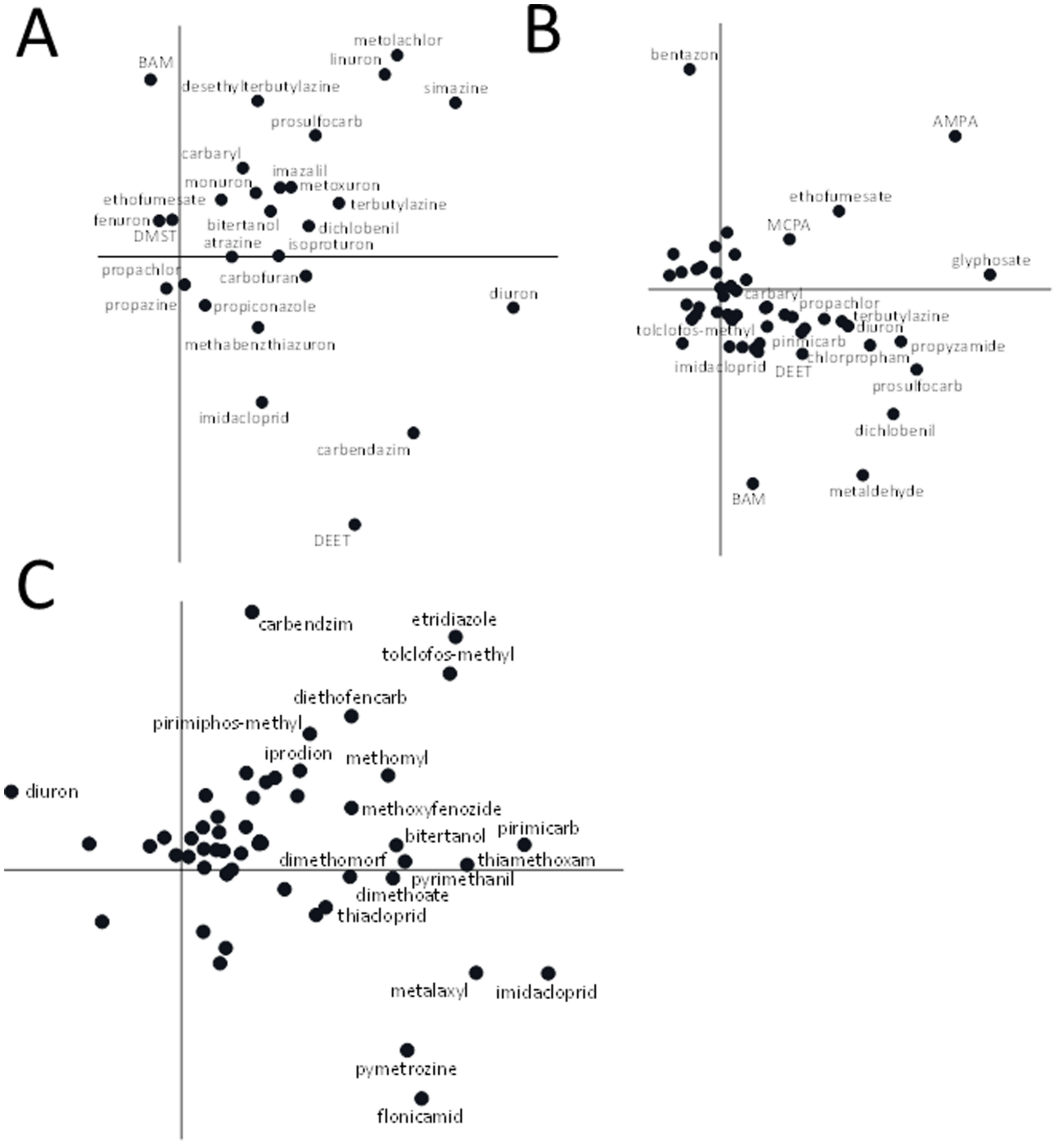
Results of the PCA analysis on data set 1 (A), 2 (B) and 3 (C). The PCA diagram of data set 1 displays 51% (33% on horizontal axis and 18% on vertical one) of the variation in chemical concentrations between the sites while 34% is displayed for data set 2 (21% on horizontal axis and 13% on vertical one) and 38% for data set 3 (28% on horizontal axis and 10% on vertical one).

Figure 3A clearly shows that imidacloprid exposure is highly correlated with all chemicals placed on the right, lower side of the diagram, like carbendazim and DEET and to a lesser extend with the large group of chemicals which have a high loading with the horizontal axis, which explains almost double the amount of variance compared to the vertical axis. The results of the second data set (Fig. 3B) show that imidacloprid is placed in the centre of a large group of pesticides placed in the middle of the diagram, since it was measured only in a few samples (7% of the total). The results of the third data set shows a high occurrence of imidacloprid above the LOR (78% of all samples), with concentrations strongly collinear with those that have a high loading on the horizontal axis which explains almost triple the amount of variance of the vertical one (Fig. 3C). The results of the first and third data set show that the contribution of imidacloprid toxicity in surface waters cannot easily be separated from the toxicity arising from other co-occurring pesticides, or indeed any other co-occurring chemical or physical stressing agent.

The correlations derived from the PCA-plots (Fig. 3) can also be explained from the fact that the active ingredient imidacloprid currently has several authorizations in 38 different products (database ctgb.nl [17], accessed 21-5-2013). The professional use ranges from the use in crops grown in glasshouses such as all different vegetables and in open systems for different bulbs of flowers, potatoes and sugarbeets. Imidacloprid is also registered for use in fruit trees including apple and pear trees. Generally, more than one pesticide is used to protect a specific crop from pest attack. Thus, depending on the land use type, imidacloprid is invariably emitted to surface waters in combination with other pesticides that are authorized to be used on those crops.

### Imidacloprid contribution in the msPAF

The potentially affected fraction of the aquatic species by the measured pesticides is higher than 5% in 11 locations (reflecting 1.2 % of all monitoring sites) in the Netherlands in the year 2009. The maximum level that we determined based on the msPAF was 23% in the province of South-Holland. Imidacloprid contributed in 8 out of 11 cases to this potential risk (Table 1). The relative contribution compared to other pesticides as measured at the same location at the same sampling time is rather modest and varied with a maximum of 21% at one location. Note that this calculation was based on acute toxicity data only, so likely is an underestimation of the potential risks that include both acute as chronic effects. From Table 1, it can be deduced that depending on location, the contribution of specific individual active ingredients differs.

**Table 1:** Contribution of imidacloprid to the msPAF at locations where msPAF > 5%.

x-coordinate	y-coordinate	Province	Total msPAF of measured pesticides (%)	Relative contribution of imidacloprid to the total msPAF of measured pesticides (%)
N 51 46 39.9	E 4 16 36.7	South Holland	22.53	0
N 52 1 29.6	E 4 30 24.7	South Holland	13.85	7.59
N 51 43 11.8	E 4 16 1.5	Zealand	12.48	0.002
N 51 52 33.5	E 4 10 26.2	South Holland	10.11	0
N 51 46 38.6	E 4 33 19.3	South Holland	9.91	0.009
N 51 45 0.4	E 4 25 46.2	South Holland	9.44	0
N 52 31 7.8	E 4 40 36.5	North Holland	9.25	0.014
N 51 57 10.2	E 4 15 8.8	South Holland	7.09	21.04
N 51 50 20	E 4 35 16.7	South Holland	6.61	0.001
N 51 21 52	E 4 2 10.1	Zealand	6.36	11.49
N 52 41 42.6	E 6 53 54.9	Drenthe	5.64	0.011

### Pairwise combinations of samples

Imidacloprid measurements performed within a time window of 160 days which were taken at sites closer than 1 km from each other were compared. By this pairwise analysis we investigate if selected pairs of imidacloprid concentrations match with each other, and subsequently can be used to accurately link biological effect data and imidacloprid concentrations. Table 2 shows that in 39% of the comparisons there was no match in the presence of imidacloprid above the LOR, while only in 23% of the cases imidacloprid was present above the LOR in both samples. The remaining 38% of comparisons showed two measurements below the LOR. So when imidacloprid is found in at least one of the samples there is a large probability (62%) of not finding imidacloprid in the other site, which hampers the extrapolation of imidacloprid over a time window of 160 day and over a distance of 1 km (Table 2). We, therefore, conclude that the criteria used by Van Dijk et al. [1] to link chemical with biological observations result in a large probability (46%) of linking a site where imidacloprid was detected with a site, where the biological sample was taken, where actually no imidacloprid could be detected. The alternative, i.e. the first measurement being below the LOR and the second one above also has a relatively high probability (34%) (Table 2). Especially in a water-rich country such as the Netherlands, that has more than 350.000 km of ditch systems [18], it should be noted that sampling locations taken within 1 km, not necessarily have a hydrological connection with each other.

**Table 2:** Result of the comparison of imidacloprid concentrations in samples taken in 2009 at sampling sites closer than 1 km and within 160 days.

Category	# sample pairs	% of total comparisons	% when 1^st^ observation is above LOR	% when 1^st^ observation is below LOR
Two below LOR	217	38		66
One below and above LOR	223	39	46	34
Two above LOR	134	23	54	
100% matching measurements	10	1.7		

LOR  =  analytical reporting limit.

### Imidacloprid dynamics

**Figure 4:**
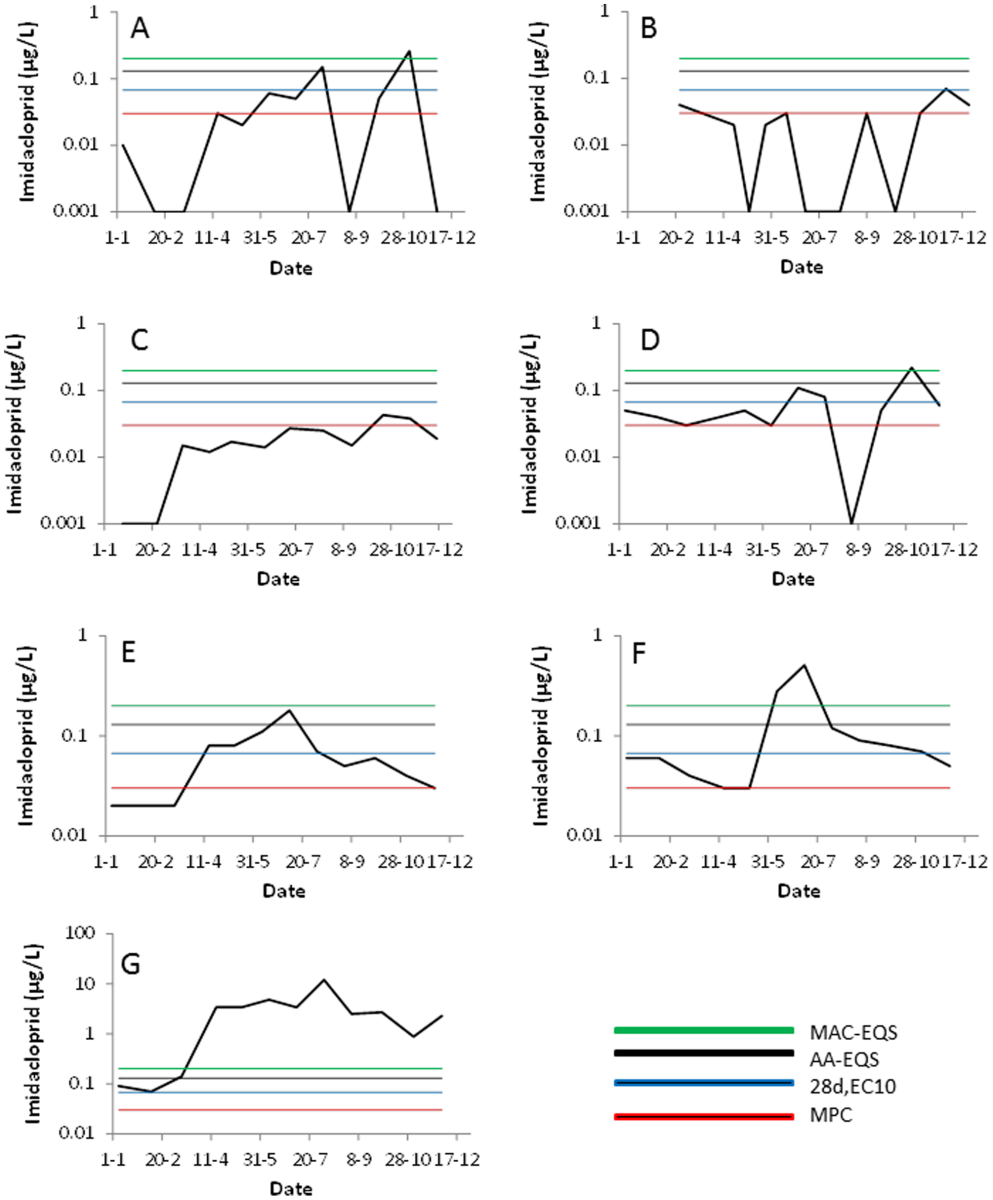
Concentration dynamics at the selected sampling sites (see text for procedure). The sampling sites 4A through 4G have X,Y coordinates of 108313,456412, 105888,455853, 103707,455196, 105927,453177, 170370,518957, 106781,503700 and 105079,453602, respectively. The horizontal lines denotes the MAC-EQS, the AA-EQS, the 28d, EC10 value for the mayflies C. horaria and C. dipterum (Roessink et al., 2013) and the MPC (top to bottom).

The concentration dynamics of imidacloprid (reflecting the concentrations of imidacloprid at the sampling locations with 10 or more samples taken in 2009 and with detection above the LOR in at least 50% of those samples) are shown in Figure 4. In all but two (Fig. 4B and 4C) of these sampling sites the 28d, EC10 values for *C. horaria* and *C. dipterum* are exceeded for a period longer than 28 days, so at these sites chronic effects of imidacloprid exposure on mayflies can be expected. Also all standards are exceeded for some time in most of the sampling sites, with Fig. 4G showing the largest exeedence for a site near Boskoop in the province of South Holland. It should be noted that these 7 sites only constitute a small percentage (18%) of the total number of sites with 10 or more observations, so likely these exposure patterns represent the worst-cases of the exposure patterns at sites with 10 or more observations. Since we don’t know whether there is a bias to measure imidacloprid more intensively at sites where exposure is expected we cannot extrapolate this to the whole population of sites.

### Maximum concentrations of imidacloprid

**Figure 5:**
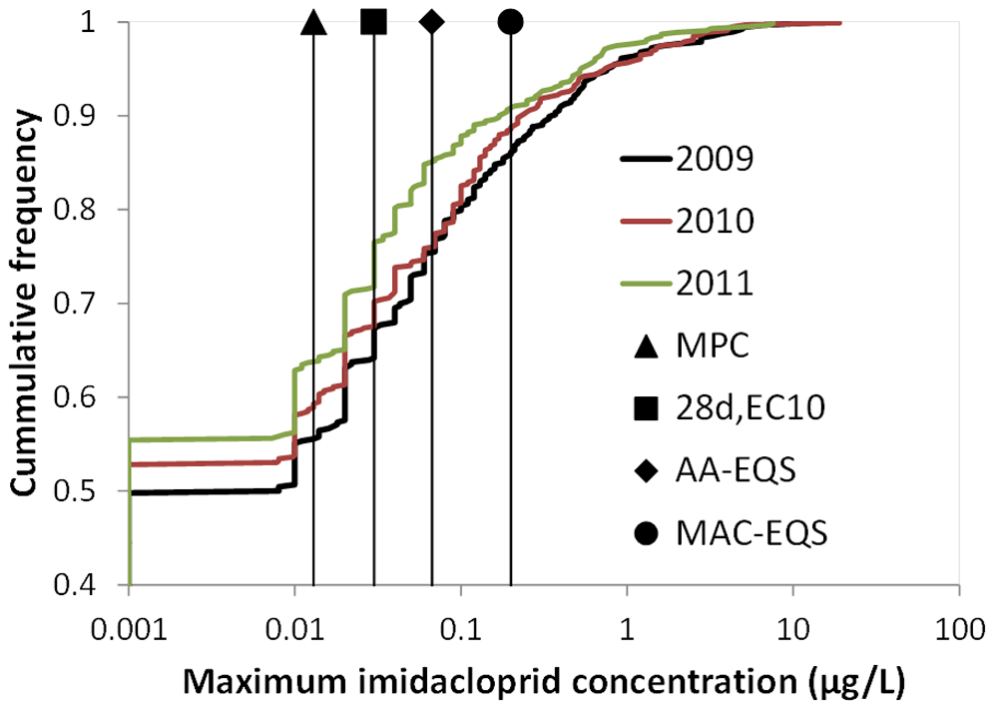
The cumulative frequency of the maximum imidacloprid concentrations of the sampling sites in 2009, 2010 and 2011, together with three standards and the 28d, EC10 of Cloeon dipterum and Caenis horaria.

Figure 5 shows the cumulative frequency of the all concentration measurements on the maximum level of imidacloprid for the years 2009, 2010 and 2011. The below LOR measurements are indicated at the 0.001 µg/L level and constituted 50, 53 and 55% of the maximum concentrations in 2009, 2010 and 2011, respectively. The results in Figure 4 show that peak concentrations of imidacloprid in the Dutch surface waters often exceeds the chronic effect concentrations of mayfly as determined in the chronic single species studies by Roessink et al. [16], as well as the three standards. In 2011 the MPC, 28d, EC10, AA-EQS and MAC-EQS threshold values are exceeded by 36, 28, 15 and 9% of the maximum concentrations at the sampling sites, respectively. Since the Hazardous Concentration 5% based on 96h,EC10 values of 0.083 µg/L [16] corresponds more or less with the AA-EQS, acute effects of imidacloprid exposure cannot be excluded at a relatively large proportion of the sites (≈15%). The maximum concentration is of course not a good predictor for the time weighted average concentration of 28d which should ideally be compared with the chronic threshold value of 0.03 µg/L. Still, when combining the results of the time-series (Fig. 3) and the exceedance of this threshold value by the maximum concentrations (Fig. 4) chronic effects of imidacloprid on insects like mayflies may be expected at a vast proportion of sites, with 28% being the most conservative estimate and 5% being the best guess. This 5% is calculated by multiplying the 28% chance of exceeding the threshold value by the maximum concentration and 15% chance of having above LOR measurements at more than 50% of the samples taken at a particular site where imidacloprid is measured at least 10 times. The comparison of the standards with the ecotoxicological threshold value for mayflies also suggests that the MAC-EQS and AA-EQS are not fully protective for acute and chronic effects on insect taxa, respectively.

### Exceedances of environmental quality standards

As stated in the Van Dijk et al [1] paper, in 2009 imidacloprid frequently exceeds quality standards for surface waters: 111 and 62 times for the AA-EQS and the MAC-EQS respectively [8], [18]. In addition to the probability of exceeding a standard, also the magnitude of exceedance is important since it is likely that at higher magnitudes the ecological effects are more severe and maybe even last longer. Table 3 shows the compounds that exceeded the MPC most frequently in 2009, ranked according to degree of exceedance.

**Table 3:** Top10 pesticides exceeding the MPC in the Netherlands in the year 2009.

Pesticides name	No. of monitoring sites	% Exceedance	No. of measurements	% Exceedance
Captan	38	47	194	13
desethyl-terbuthylazin	63	37	299	10
Imidacloprid	451	44	2133	28
Triflumuron	24	21	142	4
Dicofol	24	17	142	3
Omethoaat	31	16	169	3
Foraat	51	14	313	2
Captafol	15	27	29	14
Fipronil	69	12	230	7
Pyraclostrobin	66	17	341	7

No.  =  number. The ranking of pesticides is based on frequency and magnitude of exceedances.

Imidacloprid was predicted to have a relatively large impact on the ecosystems compared to other pesticides, and gained third place in the Top 10 pesticides violating the environmental quality standards in respect to frequency and magnitude of exceedance. The number of measurements is high, as is also the number of locations from which the samples are taken. This means that monitoring is quite intensive for this compound, and surely covers many different surface waters belonging to different water managers and covering the geographical distribution of the different water types in the Netherlands. Although less intensively measured – a factor 5 to 10 – Table 3 also shows that other pesticides exceed the MPC more often. Thus although imidacloprid poses a significant ecological risk to surface waters in the Netherlands, it is not the only potential cause of degradation in macroinvertebrate abundance, as many other pesticides mentioned in Table 3 also exceed the MPC frequently (and in cases by orders of magnitude) and thus undoubtedly contribute to overall stress regime. It is a common flaw in ecological studies to selectively interpret individual causal agents within stressor regimes as the sole cause of observed phenomena, leading to erroneous conclusions.

## Conclusion

Imidacloprid is one of several pesticides that can be detected in surface waters draining agricultural areas at levels frequently exceeding environmental quality standards. Despite this, we show here that key assumptions made by Van Dijk et al. [1] specifically relating to imidacloprid toxicity are not supported by observational data and, therefore, their assessment is unsuitable to determine threshold levels of effects. Specifically, the validity of two assumptions: 1) that imidacloprid levels are not correlated with toxic levels of other pesticides residues and 2) that chemical exposure data can be extrapolated over a 1 km distance and 160 day time window are here shown to be highly questionable. The ecological status of field sites can be attributed to a complex suite of stressors resulting from a range of anthropogenic practices in the highly managed landscape of the Netherlands, of which pesticides are just one factor, and imidacloprid only one of many pesticides being applied, albeit an important one in terms of ecological risks. We therefore propose that any risk assessment should base the ecological threshold values not solely on field observations but also largely rely on the results of controlled experiments, since these types of experiments allow a full control of separating the imidacloprid stress from other stressors.

## Supporting Information

Table S1 **Acute toxicity values of imidacloprid (source eTox database, EPA database downloaded Oct 23th 2013).** Legend: Species selected for the toxicity test were given with their scientific name and with their species group. Toxicity data were given as log10 effect concentrations at which 50% of the organisms showed adverse effects. The scientific papers from which those data are collected are given.
(DOCX)
