# Supplementary material for: Macro-Invertebrate Decline in Surface Water Polluted with Imidacloprid: A Rebuttal and Some New Analyses
Source: PLoS One. 2014 Feb 28;9(2):e89837. doi: 10.1371/journal.pone.0089837 (PMC3938502; doi:10.1371/journal.pone.0089837)
Supplement: Table S1 — Acute toxicity values of imidacloprid (source eTox database, EPA database downloaded Oct 23th 2013). Legend: Species selected for the toxicity test were given with their scientific name and with their species group. Toxicity data were given as log10 effect concentrations at which 50% of the organisms showed adverse effects. The scientific papers from which those data are collected are given. (DOCX) [file pone.0089837.s001.docx]

Table S1: Acute toxicity values of imidacloprid (source eTox database, EPA database downloaded Oct 23th 2013).

Legend: Species selected for the toxicity test were given with their scientific name and with their species group. Toxicity data were given as log10 effect concentrations at which 50% of the organisms showed adverse effects. The scientific papers from which those data are collected are given.

| Species Scientific Name | Species Group | Log10 EC50 value (μg/L) | Author | Publication Year |
| --- | --- | --- | --- | --- |
| *Asellus aquaticus* | Crustaceans | 2.903 | Lukancic,S., U. Zibrat, T. Mezek, A. Jerebic, T. Simcic, and A. Brancelj | 2010 |
| *Asellus aquaticus* | Crustaceans | 2.903 | Lukancic,S., U. Zibrat, T. Mezek, A. Jerebic, T. Simcic, and A. Brancelj | 2010 |
| *Ceriodaphnia reticulata* | Crustaceans | 3.745 | Hayasaka,D., T. Korenaga, K. Suzuki, F. Sanchez-Bayo, and K. Goka | 2012 |
| *Chydorus sphaericus* | Crustaceans | 3.167 | Sanchez-Bayo,F., and K. Goka | 2006 |
| *Chydorus sphaericus* | Crustaceans | 4.271 | Sanchez-Bayo,F., and K. Goka | 2006 |
| *Chydorus sphaericus* | Crustaceans | 3.344 | Sanchez-Bayo,F., and K. Goka | 2006 |
| *Chydorus sphaericus* | Crustaceans | 2.920 | Sanchez-Bayo,F., and K. Goka | 2006 |
| *Cypretta seurati* | Crustaceans | 1.079 | Sanchez-Bayo,F., and K. Goka | 2006 |
| *Cypretta seurati* | Crustaceans | 1.663 | Sanchez-Bayo,F., and K. Goka | 2006 |
| *Cypretta seurati* | Crustaceans | 0.000 | Sanchez-Bayo,F., and K. Goka | 2006 |
| *Cypretta seurati* | Crustaceans | 1.204 | Sanchez-Bayo,F., and K. Goka | 2006 |
| *Gammarus fossarum* | Crustaceans | 1.845 | Lukancic,S., U. Zibrat, T. Mezek, A. Jerebic, T. Simcic, and A. Brancelj | 2010 |
| *Gammarus fossarum* | Crustaceans | 1.845 | Lukancic,S., U. Zibrat, T. Mezek, A. Jerebic, T. Simcic, and A. Brancelj | 2010 |
| *Ilyocypris dentifera* | Crustaceans | 1.114 | Sanchez-Bayo,F., and K. Goka | 2006 |
| *Ilyocypris dentifera* | Crustaceans | 0.699 | Sanchez-Bayo,F., and K. Goka | 2006 |
| *Ilyocypris dentifera* | Crustaceans | 0.477 | Sanchez-Bayo,F., and K. Goka | 2006 |
| *Ilyocypris dentifera* | Crustaceans | 0.477 | Sanchez-Bayo,F., and K. Goka | 2006 |
| *Moina macrocopa* | Crustaceans | 4.656 | Hayasaka,D., T. Korenaga, K. Suzuki, F. Sanchez-Bayo, and K. Goka | 2012 |
| *Ceriodaphnia dubia* | Crustaceans; Standard Test Species | 2.757 | Hayasaka,D., T. Korenaga, K. Suzuki, F. Sanchez-Bayo, and K. Goka | 2012 |
| *Daphnia magna* | Crustaceans; Standard Test Species | 3.568 | Loureiro,S., C. Svendsen, A.L.G. Ferreira, C. Pinheiro, F. Ribeiro, and A.M.V.M. Soares | 2010 |
| *Daphnia magna* | Crustaceans; Standard Test Species | 4.636 | Hayasaka,D., T. Korenaga, K. Suzuki, F. Sanchez-Bayo, and K. Goka | 2012 |
| *Daphnia magna* | Crustaceans; Standard Test Species | 4.930 | U.S. Environmental Protection Agency, and Office of Pesticide Programs | 2013 |
| *Daphnia magna* | Crustaceans; Standard Test Species | 4.073 | Sanchez-Bayo,F., and K. Goka | 2006 |
| *Daphnia magna* | Crustaceans; Standard Test Species | 3.780 | Sanchez-Bayo,F., and K. Goka | 2006 |
| *Daphnia pulex* | Crustaceans; Standard Test Species | 4.567 | Hayasaka,D., T. Korenaga, K. Suzuki, F. Sanchez-Bayo, and K. Goka | 2012 |
| *Hyalella azteca* | Crustaceans; Standard Test Species | 1.013 | Stoughton,S.J., K. Liber, J. Culp, and A. Cessna | 2008 |
| *Cheumatopsyche brevilineata* | Insects/Spiders | 0.625 | Yokoyama,A., K. Ohtsu, T. Iwafune, T. Nagai, S. Ishihara, Y. Kobara, T. Horio, and S. Endo | 2009 |
| *Cheumatopsyche brevilineata* | Insects/Spiders | 0.686 | Yokoyama,A., K. Ohtsu, T. Iwafune, T. Nagai, S. Ishihara, Y. Kobara, T. Horio, and S. Endo | 2009 |
| *Cheumatopsyche brevilineata* | Insects/Spiders | 0.719 | Yokoyama,A., K. Ohtsu, T. Iwafune, T. Nagai, S. Ishihara, Y. Kobara, T. Horio, and S. Endo | 2009 |
| *Chironomus tentans* | Insects/Spiders; Standard Test Species | -0.041 | Stoughton,S.J., K. Liber, J. Culp, and A. Cessna | 2008 |
| *Chironomus tentans* | Insects/Spiders; Standard Test Species | 0.497 | Stoughton,S.J., K. Liber, J. Culp, and A. Cessna | 2008 |
| *Cypridopsis vidua* | Miscellaneous | 1.204 | Sanchez-Bayo,F., and K. Goka | 2006 |
| *Cypridopsis vidua* | Miscellaneous | 1.255 | Sanchez-Bayo,F., and K. Goka | 2006 |
| *Cypridopsis vidua* | Miscellaneous | 1.000 | Sanchez-Bayo,F., and K. Goka | 2006 |
| *Cypridopsis vidua* | Miscellaneous | 0.477 | Sanchez-Bayo,F., and K. Goka | 2006 |
| *Crassostrea virginica* | Molluscs; Standard Test Species | 5.161 | U.S. Environmental Protection Agency, and Office of Pesticide Programs | 2013 |
| *Lumbriculus variegatus* | Worms; Standard Test Species | 0.792 | Alexander,A.C., J.M. Culp, K. Liber, and A.J. Cessna | 2007 |
| *Tubifex tubifex* | Worms; Standard Test Species | 2.146 | Gerhardt,A. | 2009 |
| *Tubifex tubifex* | Worms; Standard Test Species | 2.041 | Gerhardt,A. | 2009 |
| *Tubifex tubifex* | Worms; Standard Test Species | 1.954 | Gerhardt,A. | 2009 |
| *Tubifex tubifex* | Worms; Standard Test Species | 1.954 | Gerhardt,A. | 2009 |
| *Pelophylax nigromaculatus* | Amphibians | 5.428 | Feng,S., Z. Kong, X. Wang, L. Zhao, and P. Peng | 2004 |
| *Pelophylax nigromaculatus* | Amphibians | 5.340 | Feng,S., Z. Kong, X. Wang, L. Zhao, and P. Peng | 2004 |
| *Pelophylax nigromaculatus* | Amphibians | 5.248 | Feng,S., Z. Kong, X. Wang, L. Zhao, and P. Peng | 2004 |
| *Pelophylax nigromaculatus* | Amphibians | 5.111 | Feng,S., Z. Kong, X. Wang, L. Zhao, and P. Peng | 2004 |
| *Rana limnocharis* | Amphibians | 5.371 | Feng,S., Z. Kong, X. Wang, L. Zhao, and P. Peng | 2004 |
| *Rana limnocharis* | Amphibians | 5.217 | Feng,S., Z. Kong, X. Wang, L. Zhao, and P. Peng | 2004 |
| *Rana limnocharis* | Amphibians | 5.064 | Feng,S., Z. Kong, X. Wang, L. Zhao, and P. Peng | 2004 |
| *Rana limnocharis* | Amphibians | 4.914 | Feng,S., Z. Kong, X. Wang, L. Zhao, and P. Peng | 2004 |
| *Artemia sp.* | Crustaceans | 5.558 | Song,M.Y., J.D. Stark, and J.J. Brown | 1997 |
| *Artemia sp.* | Crustaceans | 5.558 | Song,M.Y., and J.J. Brown | 1998 |
| *Asellus aquaticus* | Crustaceans | 3.929 | Lukancic,S., U. Zibrat, T. Mezek, A. Jerebic, T. Simcic, and A. Brancelj | 2010 |
| *Asellus aquaticus* | Crustaceans | 3.929 | Lukancic,S., U. Zibrat, T. Mezek, A. Jerebic, T. Simcic, and A. Brancelj | 2010 |
| *Callinectes sapidus* | Crustaceans | 1.002 | Osterberg,J.S. | 2010 |
| *Callinectes sapidus* | Crustaceans | 3.046 | Osterberg,J.S. | 2010 |
| *Callinectes sapidus* | Crustaceans | 2.495 | Osterberg,J.S. | 2010 |
| *Callinectes sapidus* | Crustaceans | 2.912 | Osterberg,J.S. | 2010 |
| *Callinectes sapidus* | Crustaceans | 1.002 | Osterberg,J.S., K.M. Darnell, T.M. Blickley, J.A. Romano, and D. Rittschof | 2012 |
| *Callinectes sapidus* | Crustaceans | 3.046 | Osterberg,J.S., K.M. Darnell, T.M. Blickley, J.A. Romano, and D. Rittschof | 2012 |
| *Callinectes sapidus* | Crustaceans | 2.495 | Osterberg,J.S., K.M. Darnell, T.M. Blickley, J.A. Romano, and D. Rittschof | 2012 |
| *Callinectes sapidus* | Crustaceans | 2.912 | Osterberg,J.S., K.M. Darnell, T.M. Blickley, J.A. Romano, and D. Rittschof | 2012 |
| *Chydorus sphaericus* | Crustaceans | 5.209 | Sanchez-Bayo,F., and K. Goka | 2006 |
| *Chydorus sphaericus* | Crustaceans | 5.123 | Sanchez-Bayo,F., and K. Goka | 2006 |
| *Cypretta seurati* | Crustaceans | 2.865 | Sanchez-Bayo,F., and K. Goka | 2006 |
| *Cypretta seurati* | Crustaceans | 2.479 | Sanchez-Bayo,F., and K. Goka | 2006 |
| *Gammarus fossarum* | Crustaceans | 2.903 | Lukancic,S., U. Zibrat, T. Mezek, A. Jerebic, T. Simcic, and A. Brancelj | 2010 |
| *Gammarus fossarum* | Crustaceans | 1.845 | Lukancic,S., U. Zibrat, T. Mezek, A. Jerebic, T. Simcic, and A. Brancelj | 2010 |
| *Gammarus pulex* | Crustaceans | 3.943 | Ashauer,R., I. Caravatti, A. Hintermeister, and B.I. Escher | 2010 |
| *Gammarus pulex* | Crustaceans | 3.586 | Ashauer,R., I. Caravatti, A. Hintermeister, and B.I. Escher | 2010 |
| *Gammarus pulex* | Crustaceans | 2.431 | Beketov,M.A., and M. Liess | 2008 |
| *Hyalella sp.* | Crustaceans | 1.740 | U.S. Environmental Protection Agency, and Office of Pesticide Programs | 2013 |
| *Ilyocypris dentifera* | Crustaceans | 3.050 | Sanchez-Bayo,F., and K. Goka | 2006 |
| *Ilyocypris dentifera* | Crustaceans | 2.880 | Sanchez-Bayo,F., and K. Goka | 2006 |
| *Ilyocypris dentifera* | Crustaceans | 2.330 | Sanchez-Bayo,F., and K. Goka | 2006 |
| *Ilyocypris dentifera* | Crustaceans | 2.713 | Sanchez-Bayo,F., and K. Goka | 2006 |
| *Palaemonetes pugio* | Crustaceans | 2.490 | Key,P., K. Chung, T. Siewicki, and M. Fulton | 2007 |
| *Palaemonetes pugio* | Crustaceans | 2.751 | Key,P., K. Chung, T. Siewicki, and M. Fulton | 2007 |
| *Americamysis bahia* | Crustaceans; Standard Test Species | 1.580 | U.S. Environmental Protection Agency, and Office of Pesticide Programs | 2013 |
| *Americamysis bahia* | Crustaceans; Standard Test Species | 2.201 | U.S. Environmental Protection Agency, and Office of Pesticide Programs | 2013 |
| *Daphnia magna* | Crustaceans; Standard Test Species | 4.987 | Loureiro,S., C. Svendsen, A.L.G. Ferreira, C. Pinheiro, F. Ribeiro, and A.M.V.M. Soares | 2010 |
| *Daphnia magna* | Crustaceans; Standard Test Species | 5.505 | Sanchez-Bayo,F., and K. Goka | 2006 |
| *Daphnia magna* | Crustaceans; Standard Test Species | 4.019 | Song,M.Y., J.D. Stark, and J.J. Brown | 1997 |
| *Daphnia magna* | Crustaceans; Standard Test Species | 4.240 | Song,M.Y., J.D. Stark, and J.J. Brown | 1997 |
| *Daphnia magna* | Crustaceans; Standard Test Species | 4.812 | Sanchez-Bayo,F., and K. Goka | 2006 |
| *Hyalella azteca* | Crustaceans; Standard Test Species | 0.846 | Stoughton,S.J., K. Liber, J. Culp, and A. Cessna | 2008 |
| *Hyalella azteca* | Crustaceans; Standard Test Species | 0.850 | Stoughton,S.J., K. Liber, J. Culp, and A. Cessna | 2008 |
| *Hyalella azteca* | Crustaceans; Standard Test Species | 1.242 | Stoughton,S.J., K. Liber, J. Culp, and A. Cessna | 2008 |
| *Hyalella azteca* | Crustaceans; Standard Test Species | 1.816 | Stoughton,S.J., K. Liber, J. Culp, and A. Cessna | 2008 |
| *Hyalella azteca* | Crustaceans; Standard Test Species | 2.062 | U.S. Environmental Protection Agency, and Office of Pesticide Programs | 2013 |
| *Cyprinodon variegatus* | Fish; Standard Test Species | 5.212 | U.S. Environmental Protection Agency, and Office of Pesticide Programs | 2013 |
| *Lepomis macrochirus* | Fish; Standard Test Species | 5.021 | U.S. Environmental Protection Agency, and Office of Pesticide Programs | 2013 |
| *Oncorhynchus mykiss* | Fish; Standard Test Species; U.S. Threatened and Endangered Species | 4.919 | U.S. Environmental Protection Agency, and Office of Pesticide Programs | 2013 |
| *Oncorhynchus mykiss* | Fish; Standard Test Species; U.S. Threatened and Endangered Species | 5.360 | U.S. Environmental Protection Agency, and Office of Pesticide Programs | 2013 |
| *Aedes aegypti* | Insects | 1.568 | Pridgeon,J.W., J.J. Becnel, G.G. Clark, and K.J. Linthicum | 2009 |
| *Aedes aegypti* | Insects | 2.914 | Riaz,M.A., R. Poupardin, S. Reynaud, C. Strode, H. Ranson, and J.P. David | 2009 |
| *Aedes aegypti* | Insects | 1.643 | Song,M.Y., J.D. Stark, and J.J. Brown | 1997 |
| *Aedes aegypti* | Insects | 1.653 | Song,M.Y., J.D. Stark, and J.J. Brown | 1997 |
| *Aedes aegypti* | Insects | 1.924 | Paul,A., L.C. Harrington, and J.G. Scott | 2006 |
| *Aedes taeniorhynchus* | Insects | 1.114 | Song,M.Y., J.D. Stark, and J.J. Brown | 1997 |
| *Aedes taeniorhynchus* | Insects | 1.114 | Song,M.Y., and J.J. Brown | 1998 |
| *Aedes taeniorhynchus* | Insects | 1.322 | Song,M.Y., and J.J. Brown | 1998 |
| *Baetis rhodani* | Insects | 0.929 | Beketov,M.A., and M. Liess | 2008 |
| *Chironomus dilutus* | Insects | 0.423 | LeBlanc,H.M.K., J.M. Culp, D.J. Baird, A.C. Alexander, and A.J. Cessna | 2012 |
| *Culex quinquefasciatus* | Insects | 2.301 | Liu,H., E.W. Cupp, K.M. Micher, A. Guo, and N. Liu | 2004 |
| *Culex quinquefasciatus* | Insects | 2.477 | Liu,H., E.W. Cupp, K.M. Micher, A. Guo, and N. Liu | 2004 |
| *Culex quinquefasciatus* | Insects | 1.602 | Liu,H., E.W. Cupp, K.M. Micher, A. Guo, and N. Liu | 2004 |
| *Culex quinquefasciatus* | Insects | 2.602 | Liu,H., E.W. Cupp, K.M. Micher, A. Guo, and N. Liu | 2004 |
| *Epeorus longimanus* | Insects | 0.322 | Alexander,A.C., J.M. Culp, K. Liber, and A.J. Cessna | 2007 |
| *Epeorus longimanus* | Insects | 0.322 | Alexander,A.C., J.M. Culp, K. Liber, and A.J. Cessna | 2007 |
| *Epeorus longimanus* | Insects | -0.187 | Alexander,A.C., J.M. Culp, K. Liber, and A.J. Cessna | 2007 |
| *Pteronarcys dorsata* | Insects | 1.846 | Kreutzweiser,D.P., K.P. Good, D.T. Chartrand, T.A. Scarr, and D.G. Thompson | 2008 |
| *Simulium latigonium* | Insects | 0.572 | Beketov,M.A., and M. Liess | 2008 |
| *Simulium vittatum* | Insects | 0.829 | Overmyer,J.P., B.N. Mason, and K.L. Armbrust | 2005 |
| *Simulium vittatum* | Insects | 0.916 | Overmyer,J.P., B.N. Mason, and K.L. Armbrust | 2005 |
| *Simulium vittatum* | Insects | 0.980 | Overmyer,J.P., B.N. Mason, and K.L. Armbrust | 2005 |
| *Tipula sp.* | Insects | 2.143 | Kreutzweiser,D.P., K.P. Good, D.T. Chartrand, T.A. Scarr, and D.G. Thompson | 2008 |
| *Chironomus sp.* | Insects; Standard Test Species | 1.838 | U.S. Environmental Protection Agency, and Office of Pesticide Programs | 2013 |
| *Chironomus tentans* | Insects; Standard Test Species | 0.732 | Stoughton,S.J., K. Liber, J. Culp, and A. Cessna | 2008 |
| *Chironomus tentans* | Insects; Standard Test Species | 0.760 | Stoughton,S.J., K. Liber, J. Culp, and A. Cessna | 2008 |
| *Aedes albopictus* | Insects; U.S. Exotic/ Nuisance Species | 2.477 | Liu,H., E.W. Cupp, A. Guo, and N. Liu | 2004 |
| *Aedes albopictus* | Insects; U.S. Exotic/ Nuisance Species | 2.699 | Liu,H., E.W. Cupp, A. Guo, and N. Liu | 2004 |
| *Aedes albopictus* | Insects; U.S. Exotic/ Nuisance Species | 2.778 | Liu,H., E.W. Cupp, A. Guo, and N. Liu | 2004 |
| *Aedes albopictus* | Insects; U.S. Exotic/ Nuisance Species | 2.778 | Liu,H., E.W. Cupp, A. Guo, and N. Liu | 2004 |
| *Aedes albopictus* | Insects/Spiders; U.S. Exotic/ Nuisance Species | 2.903 | Liu,H., E.W. Cupp, A. Guo, and N. Liu | 2004 |
| *Cypridopsis vidua* | Miscellaneous | 3.602 | Sanchez-Bayo,F., and K. Goka | 2006 |
| *Cypridopsis vidua* | Miscellaneous | 2.436 | Sanchez-Bayo,F., and K. Goka | 2006 |
| *Cypridopsis vidua* | Miscellaneous | 2.854 | Sanchez-Bayo,F., and K. Goka | 2006 |
| *Agamermis unka* | Worms | 3.199 | Choo,H.Y., H.H. Kim, and H.K. Kaya | 1998 |
| *Tubifex tubifex* | Worms; Standard Test Species | 2.505 | Gerhardt,A. | 2009 |
